# The Comparative Impact of Liberal Versus Conservative Oxygenation in Critically Ill COVID-19 Patients: A Retrospective Study

**DOI:** 10.7759/cureus.67809

**Published:** 2024-08-26

**Authors:** Deepak Singla, Priya TK, Anirban B Adhikary, Dhatri Jonna, Mishu Mangla

**Affiliations:** 1 Anesthesiology and Critical Care, All India Institute of Medical Sciences, Rishikesh, Rishikesh, IND; 2 Anesthesiology and Critical Care, All India Institute of Medical Sciences, Jodhpur, Jodhpur, IND; 3 Obstetrics and Gynecology, All India Institute of Medical Sciences, Bibinagar, Hyderabad, IND

**Keywords:** glasgow coma scale, acute kidney injury, lactates, sequential organ failure assessment score, covid-19, oxygen inhalation therapy

## Abstract

Objectives

Whether a higher or lower partial pressure of oxygen (PaO_2_) could impact outcomes in patients with coronavirus disease 2019 (COVID-19) remains a matter of debate. So, we planned this retrospective analysis to determine if a higher or lower partial pressure of oxygen in blood had any effect on outcomes in COVID-19 patients.

Material and method

The records of COVID-19 patients from the beginning of 2020 to the end of 2022 were scanned. Patients were sub-grouped into two groups based on the partial pressure of oxygen (PaO_2_) values on arterial blood gas (ABG), i.e., high PaO_2 _group, PaO_2_ value of 80-100 mm Hg, and low PaO_2 _group, PaO_2_ value of 60-80 mm Hg for the first 48 hours after the initiation of oxygenation and/or mechanical ventilation. The two groups were compared in terms of partial pressure of oxygen in arterial blood to the fraction of inspiratory oxygen (FiO_2_) concentration (P/F ratio), Sequential Organ Failure Assessment (SOFA) score at presentation and after 48 hours, and clinical outcomes, including mortality, time of mortality, extubation, acute kidney injury (AKI), and change in Glasgow Coma Scale (GCS).

Results

SOFA score was significantly higher in the low PaO_2_ group as compared to the high PaO_2_ group both at baseline (4.59 {1.79} versus 5.51 {1.15}; p-value: 0.005) and at 48 hours (3.06 {1.39} versus 5.11 {2.13}; p-value: 0.007). However, the change in SOFA score over 48 hours did not achieve statistical significance (-1.000 {0.97} versus 0.53 {2.34}; p-value: 0.257). Out of a total of 37 patients, 21 patients died in the high PaO_2_ group, while 18 patients died in the low PaO_2_ group.

Conclusion

Our study highlights that targeting either low or high arterial oxygen content while considering oxygen therapy for COVID-19 patients did not significantly alter the outcomes.

## Introduction

Given oxygen's integral role in sustaining our metabolism, its function becomes even more essential in states of limited supply such as coronavirus disease 2019 (COVID-19) infection. However, the PROXI [1] trial failed to demonstrate any benefit of a high inspired oxygen fraction on surgical site infection. In fact, the mortality was found to be higher in patients receiving higher concentrations of inspired oxygen. Another study demonstrated a typical "U"-shaped relationship between partial pressure of oxygen (PaO_2_) and mortality [2]. Both high and low PaO_2_ values were associated with higher mortality. The pathophysiology behind the harmful effects of high inspired oxygen concentration, or hyperoxia, is the increased production of reactive oxygen species (ROS), absorption atelectasis, and reduction in ventilation, among others [2].

The problem of supplemental oxygenation is compounded by the uncertainty reported by a high concentration of oxygen delivered as therapy and its safety in various studies. These questions and doubts have led to disarray as there has yet to be a consensus on a standardized protocol for oxygenation targets in critically ill patients. With the advent of newer modalities of oxygen delivery, such as continuous positive airway pressure (CPAP), noninvasive positive pressure ventilation (NIPPV), and high-flow nasal oxygen (HFNO), oxygen concentration can be titrated precisely, allowing a much deeper understanding of the human respiratory system and its response to different concentrations of oxygen.

COVID-19 causes injury to lung vasculature, resulting in the loss of alveolar function. Various studies have mentioned that oxidative stress plays a vital role in the pathogenesis of COVID-19 [3] as it is the principal mechanism of defense against the virus. Studies have found that there was a moderately higher risk of severe COVID-19 in patients with respiratory diseases such as chronic obstructive pulmonary disease (COPD) and interstitial lung diseases that can result in low PaO_2_ [4]. However, their risk of death was lower than that of the general population.

So, whether a higher or lower partial pressure of oxygen could impact outcomes in patients with COVID-19 remains a matter of debate. So, we planned this retrospective analysis to determine whether higher or lower partial pressure of oxygen in blood had any effect on outcomes in COVID-19 patients.

## Materials and methods

After obtaining approval from the Institutional Ethics Committee of the All India Institute of Medical Sciences, Rishikesh (vide letter number AIIMS/IEC/24/297), we assessed and analyzed the records of COVID-19 patients based on the following inclusion and exclusion criteria. Records of male and female patients, aged >18 years, with COVID-19 requiring oxygen or ventilatory support with a PaO_2_ value between 60 and 100 mm Hg during the first 48 hours of admission were considered for this study. Records with PaO_2_ values above or below the desired range, a history of severe chronic obstructive pulmonary disease (COPD) (Global Initiative for Chronic Obstructive Lung Disease {GOLD} class 3 and 4), severe acute respiratory distress syndrome (ARDS) with PaO_2_/fraction of inspiratory oxygen (FiO_2_) of <100 on arterial blood gas (ABG), cardiac disease (ischemic heart disease, valvular heart disease, congestive heart failure, etc.), sickle cell disease, immunosuppressed patient or moderate to severe neutropenia (absolute neutrophil count of <1000) or post solid organ transplant, acute kidney injury (AKI), or acute liver disease (ALD) on admission were excluded from our study.

Records of COVID-19 patients from the beginning of year 2020 to the end of year 2022 were scanned. Patients were sub-grouped into two groups based on the PaO_2_ values on ABG, i.e., high PaO_2 _group, PaO_2_ value of 80-100 mm Hg, and low PaO_2 _group, PaO_2_ value of 60-80 mm Hg for the first 48 hours after the initiation of oxygenation and/or mechanical ventilation. The two groups were compared in terms of the demographic profile of the patients, comorbidities, baseline PaO_2_ values, fraction of oxygen (FiO_2_), partial pressure of oxygen in arterial blood to the fraction of inspiratory oxygen concentration (P/F ratio) during first 48 hours, lactate level during first 48 hours, Sequential Organ Failure Assessment (SOFA) score at presentation and after 48 hours, and clinical outcomes during first 48 hours. Clinical outcomes, including mortality within 48 hours, the time of mortality, extubation within 48 hours, acute kidney injury (AKI), and change in Glasgow Coma Scale (GCS), were analyzed.

The primary objective of our study was to compare the difference in the SOFA score from baseline to 48 hours. The secondary objectives included mortality within the first 48 hours, extubation within the first 48 hours, the incidence of AKI, and change in GCS in both groups at 48 hours.

Sample size

Since there are no previous studies, we included data from all the records that were consistent with our inclusion and exclusion criteria.

Statistical analysis

Data was coded and recorded in MS Excel (Microsoft Corp., Redmond, WA) spreadsheet program. Statistical Package for Social Sciences (SPSS) v23 (IBM SPSS Statistics, Armonk, NY) was used for data analysis. Descriptive statistics were elaborated on as means/standard deviations (SD), medians/IQRs for continuous variables, and frequencies/percentages for categorical variables. For normally distributed quantitative data, an unpaired "t" test was used, and for non-normally distributed data, we used the Whitney "U" test. For categorical data, a chi-squared (χ^2^) test or Fisher's exact test was used. A Kaplan-Meier curve with a log-rank test was used to assess mortality over time.

## Results

**Figure 1:**
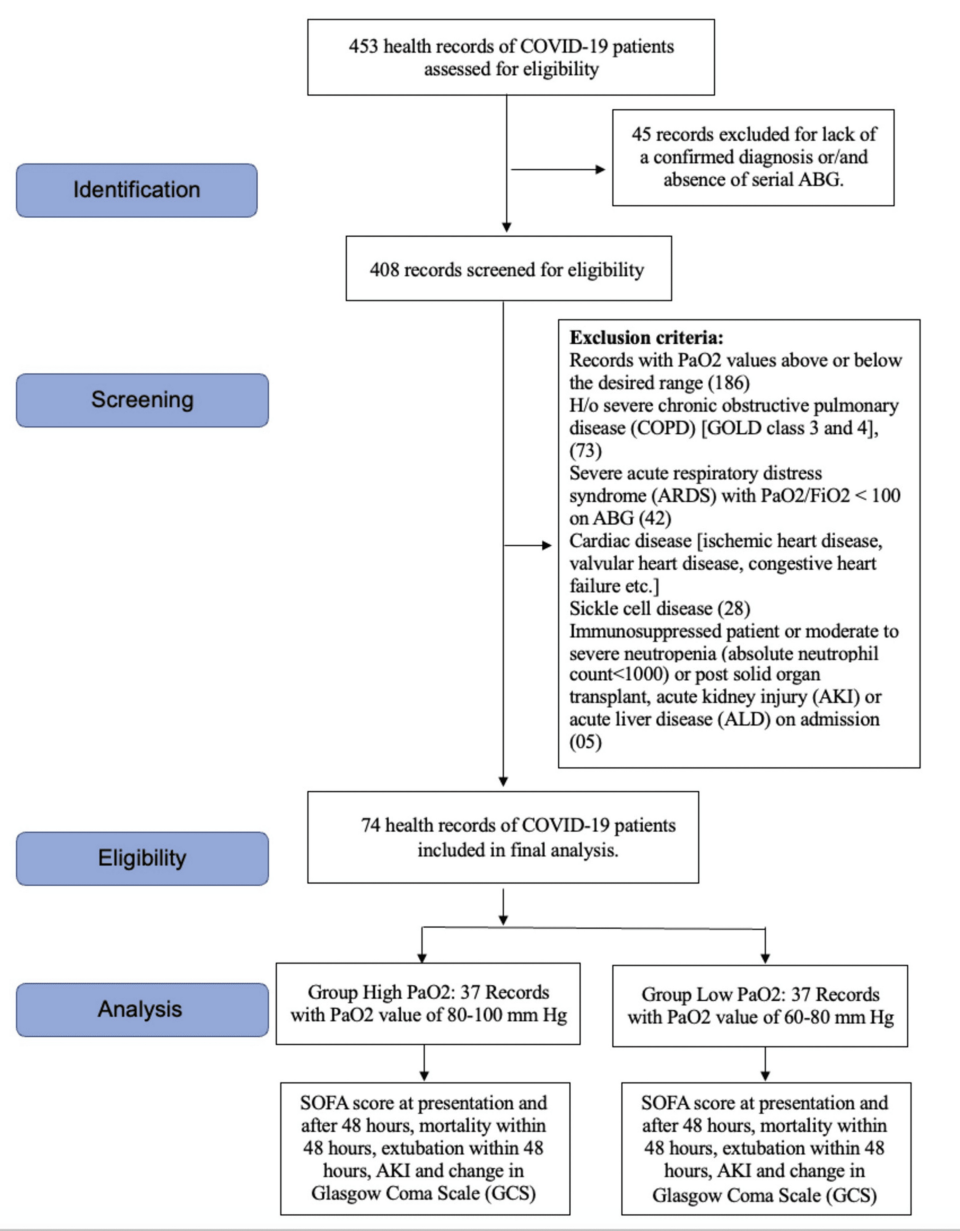
Strengthening the Reporting of Observational Studies in Epidemiology (STROBE) flow diagram COVID-19, coronavirus disease 2019; GOLD, Global Initiative for Chronic Obstructive Lung Disease; SOFA, Sequential Organ Failure Assessment; ABG, arterial blood gas; PaO_2_, partial pressure of oxygen; FiO_2_, fraction of inspiratory oxygen; h/o, history of

We analyzed the records of 453 COVID-19 patients for our study. Out of these, 45 records were excluded in the initial screening as either the diagnosis was not confirmed or serial ABG values were not there (Figure 1). Of the remaining 408 patients, 334 records were excluded based on the exclusion criteria, so we could include 74 patient records in the final analysis (Figure 1). Based on the PaO_2_ values, these patients were divided into high PaO_2_ (37 patients) and low PaO_2_ groups (37 patients) (Figure 1).

The demographic profile of the patients was comparable in both groups (Table 1). All the patients included in our analysis received mechanical ventilation (either intubated or noninvasive via facemask), and no patient was on high-flow nasal cannula or only oxygen supplementation. The relative number of patients in each group receiving invasive mechanical ventilation versus noninvasive ventilation is compared in Table 1.

**Table 1:** Baseline demographic and clinical profile of the patients in the two groups ᵗT test ᵐMann-Whitney U test ᶠFisher's exact test ^c^Chi-squared (χ^2^) test SD, standard deviation; bpm, beats per minute; PaO_2_, partial pressure of oxygen

Variable	Parameter	Total	Group		Difference (95% CI)	Significance
			High PaO_2_	Low PaO_2_		
Age (years)	Mean ± SD	55.05 ± 13.11	54.46 ± 11.11	55.65 ± 14.97	-1.19 (-7.31 to 4.93)	t = -0.388 and p = 0.699ᵗ
Age	18-30 years	2 (2.70%)	0 (0.00%)	2 (5.41%)	-5.41% (-100.00% to 1.88%)	χ² = 0.514 and p = 0.493ᶠ
	31-40 years	7 (9.46%)	3 (8.11%)	4 (10.81%)	-2.70% (-100.00% to 10.62%)	χ² = 0.000 and p = 1.000ᶠ
	41-50 years	18 (24.32%)	12 (32.43%)	6 (16.22%)	16.22% (-100.00% to 35.41%)	χ² = 1.835 and p = 0.175ᶠ
	51-60 years	17 (22.97%)	9 (24.32%)	8 (21.62%)	2.70% (-100.00% to 21.86%)	χ² = 0.000 and p = 1.000ᶠ
	61-70 years	23 (31.08%)	11 (29.73%)	12 (32.43%)	-2.70% (-100.00% to 18.38%)	χ² = 0.000 and p = 1.000ᶠ
	71-80 years	7 (9.46%)	2 (5.41%)	5 (13.51%)	-8.11% (-100.00% to 5.10%)	χ² = 0.631 and p = 0.430ᶠ
Gender	Male	43 (58.11%)	23 (62.16%)	20 (54.05%)	8.11% (-100.00% to 30.52%)	χ² = 0.222 and p = 0.638ᶠ
	Female	31 (41.89%)	14 (37.84%)	17 (45.95%)	-8.11% (-100.00% to 14.30%)	χ² = 0.222 and p = 0.638ᶠ
Any comorbidities	Yes	56 (75.68%)	29 (78.38%)	27 (72.97%)	5.41% (-100.00% to 24.92%)	χ² = 0.073 and p = 0.787ᶠ
	No	18 (24.32%)	8 (21.62%)	10 (27.03%)	-5.41% (-100.00% to 14.11%)	χ² = 0.073 and p = 0.787ᶠ
Respiratory rate (bpm) at presentation	Mean ± SD	24.58 ± 6.93	23.49 ± 6.55	25.74 ± 7.23	-2.26 (-5.51 to 0.99)	t = -1.386 and p = 0.170ᵗ
	Median (IQR)	24.00 (19.50 to 28.00)	24.00 (18.00 to 28.00)	24.00 (20.50 to 31.50)		
Lactate (mmol/L) (baseline)	Mean ± SD	3.19 ± 2.58	2.56 ± 1.89	3.81 ± 3.01	-1.26 (-2.43 to -0.09)	W = 518.500 and p = 0.073ᵐ
Mechanical ventilation	Invasive	53 (71.62%)	30 (81.08%)	23 (62.16%)		χ² = 3.258 and p = 0.071^c^
	Noninvasive	21 (28.37%)	07 (18.91%)	14 (37.83%)		

**Figure 2:**
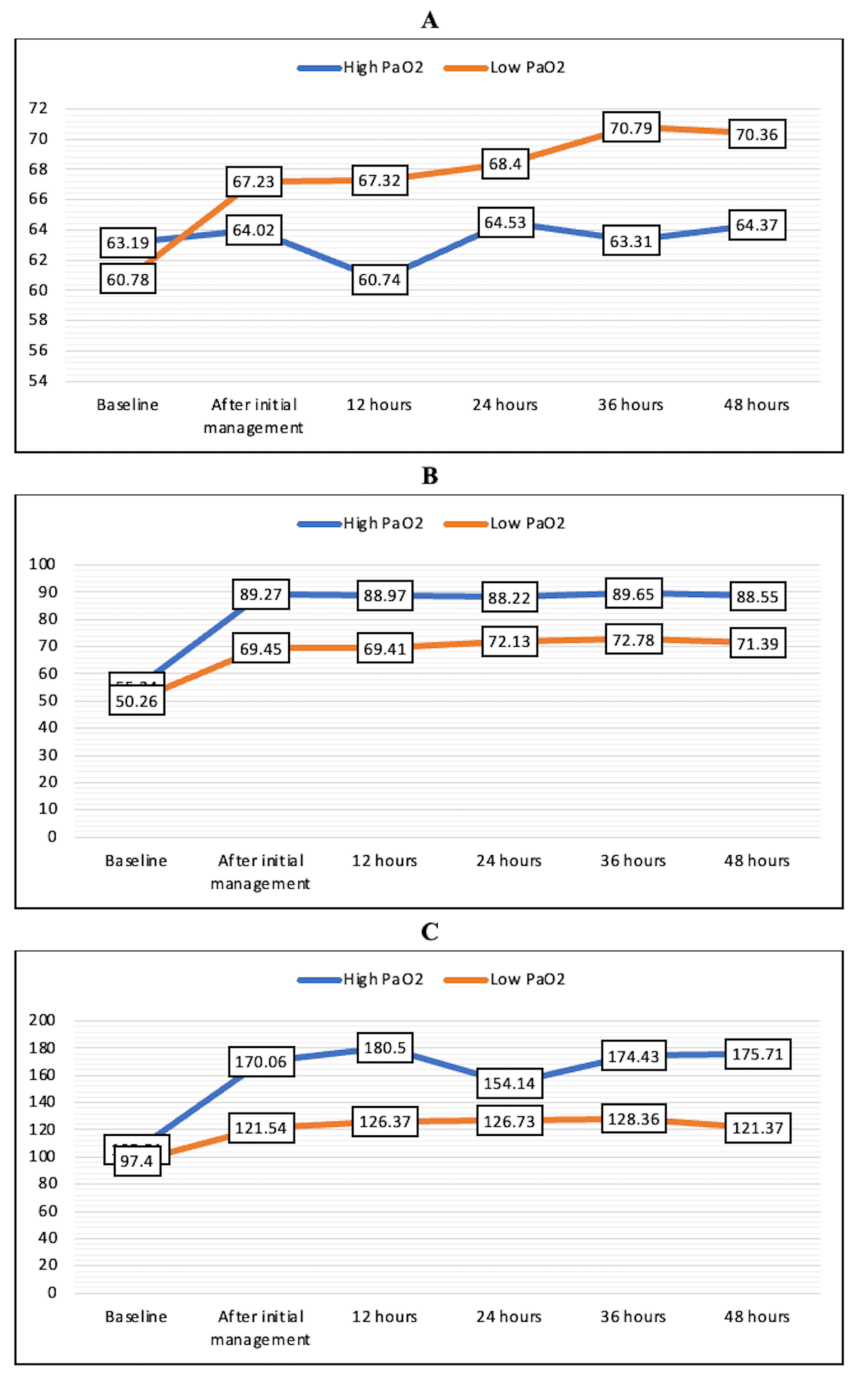
Mean FiO2 (A), PaO2 (B), and PaO2/FiO2 (C) in the high PaO2 and low PaO2 groups over the first 48 hours FiO_2_, fraction of inspiratory oxygen; PaO_2_, partial pressure of oxygen

The mean FiO_2_ requirement was higher in the low PaO_2_ group at all time points during the first 48 hours, but the difference was not statistically significant (Figure 2A). PaO_2_ values were significantly lower in the low PaO_2_ group at all time points except the baseline value (Figure 2B). The PaO_2_/FiO_2_ ratio was comparable in both groups at all time points, and no statistically significant difference was noted, although the mean values were higher in the high PaO_2_ group (Figure 2C).

The lactate levels were also comparable at all time points (Table 2).

**Table 2:** Serum lactate (mmol/L) levels in the two groups over the first 48 hours SD, standard deviation; PaO_2_, partial pressure of oxygen

Lactate (mmol/L)	Group		P-value (Wilcoxon-Mann-Whitney test)
	High PaO_2_	Low PaO_2_	
	Mean (SD)	Mean (SD)	
Baseline	2.56 (1.89)	3.81 (3.01)	0.073
After initial management	4.74 (4.49)	4.65 (3.57)	0.669
12 hours	4.70 (4.16)	4.26 (3.98)	0.685
24 hours	5.21 (5.45)	5.48 (4.24)	0.390
36 hours	4.03 (5.24)	5.17 (5.34)	0.384
48 hours	1.95 (1.03)	2.73 (1.98)	0.288

SOFA score was significantly higher in the low PaO_2_ group as compared to the high PaO_2_ group at baseline and 48 hours (Table 3). However, the change in SOFA score over 48 hours did not achieve statistical significance (Table 3).

**Table 3:** Comparison of the two groups in terms of change in SOFA over 48 hours SD, standard deviation; PaO2, partial pressure of oxygen; SOFA, Sequential Organ Failure Assessment

SOFA	Group		P-value (Wilcoxon-Mann-Whitney Test)
	High PaO_2_	Low PaO_2_	
	Mean (SD)	Mean (SD)	
Baseline	4.59 (1.79)	5.51 (1.15)	0.005
48 hours	3.06 (1.39)	5.11 (2.13)	0.007
Change in SOFA	-1.000 (0.97)	0.53 (2.34)	0.257

**Figure 3:**
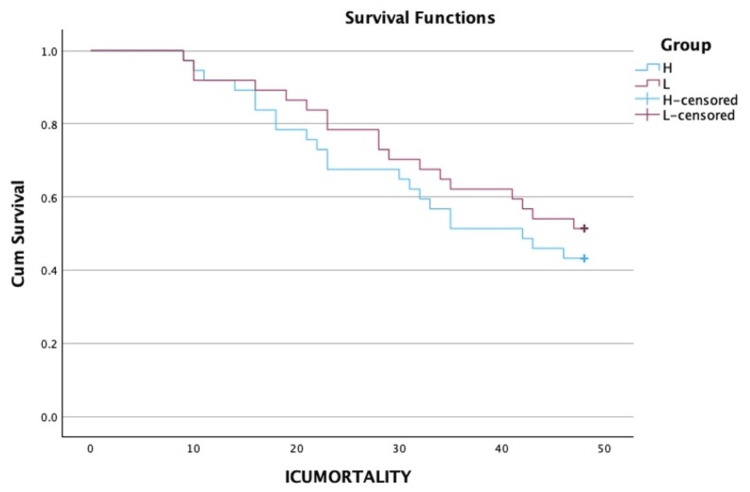
A Kaplan-Meier curve with a log-rank test ICU: intensive care unit

There was no significant difference between the various groups in terms of the distribution of two-day intensive care unit (ICU) mortality (χ^2^ = 0.487 and p = 0.485) (Table 4). In terms of the time of ICU mortality (hours), the mean (SD) for the high PaO_2_ group was 25.14 (11.22), and in the low PaO_2_ group, it was 27.22 (11.82). The median (IQR) for the time of mortality (in hours) in the high PaO_2_ group was 23 (16-34), and in the low PaO_2_ group, it was 28 (19-35). Applying an unpaired t test, the p-value came to be 0.579 (not significant). A Kaplan-Meier curve with a log-rank test for the same is given in Figure 3. Thirty patients in the high PaO_2_ group required intubation, out of which seven were extubated in the first 48 hours. Similarly, 23 patients required intubation in the low paO_2_ group, of which two were extubated in the first 48 hours (Table 4). Fisher's exact test was used to explore the association between "group" and "extubation" in the first 48 hours, as more than 20% of the total number of cells had an expected count of less than five. There was no significant difference between the various groups in terms of the distribution of extubation (48 hours) (χ^2^ = 1.979 and p = 0.270) (Table 4). The strength of association between the two variables (Cramér's V) = 0.19 (low association). The strength of association between the two variables (bias-corrected Cramér's V) = 0.14 (low association). The incidence of AKI over the first 48 hours was also comparable in both groups (χ^2^ = 0.058 and p = 0.809) (Table 4). There was no significant difference between the various groups regarding the distribution of change in GCS over 48 hours (χ^2^ = 1.367 and p = 0.242) (Table 4).

**Table 4:** Comparison of the two groups in terms of mortality, extubation, the incidence of AKI, and change in GCS over the first 48 hours PaO_2_, partial pressure of oxygen; AKI, acute kidney injury; GCS, Glasgow Coma Scale

Mortality in the first 48 hours (A)	Group			Chi-squared (χ^2^) test	
	High PaO_2_	Low PaO_2_	Total	χ^2^	P-value
Yes	21 (56.8%)	18 (56.8%)	39 (56.8%)	0.487	0.485
No	16 (43.2%)	19 (43.2%)	35 (43.2%)		
Total	37 (100.0%)	37 (100.0%)	74 (100.0%)		
Extubation in the first 48 hours (B)				Fisher's exact test	
Yes	7 (23.3%)	2 (8.7%)	9 (17.0%)	1.979	0.270
No	23 (76.7%)	21 (91.3%)	44 (83.0%)		
Total	30 (100.0%)	23 (100.0%)	53 (100.0%)		
Incidence of AKI over 48 hours (C)				Chi-squared test	
Yes	24 (64.9%)	23 (62.2%)	47 (63.5%)	0.058	0.809
No	13 (35.1%)	14 (37.8%)	27 (36.5%)		
Total	37 (100.0%)	37 (100.0%)	74 (100.0%)		
Change in GCS over 48 hours (D)				Chi-squared test	
Yes	19 (51.4%)	14 (37.8%)	33 (44.6%)	1.367	0.242
No	18 (48.6%)	23 (62.2%)	41 (55.4%)		
Total	37 (100.0%)	37 (100.0%)	74 (100.0%)		

## Discussion

In this retrospective study, we were able to analyze the records of 74 patients with 37 patients in both high and low PaO_2_ groups. The mean FiO_2_ requirements were less, and the PaO_2_/FiO_2_ ratio was slightly higher in the high PaO_2_ group, but the difference was not statistically significant at any time point. Lactate levels were also comparable in both groups. Similarly, though SOFA scores were significantly higher in the low PaO_2_ group at baseline and at 48 hours, the change in SOFA score did not achieve statistical significance. Though more patients died in the high PaO_2_ group as compared to the low PaO_2_ group, there was no statistically significant difference in terms of ICU mortality at 48 hours between the two groups. We also found a low association between PaO_2_ value and the incidence of extubation. The incidence of AKI and change in GCS were also not significantly different between the two groups.

We compared the FiO_2_ requirements and PaO_2_/FiO_2_ ratio in both the patient subsets and found a lower requirement of FiO_2_ and a higher PaO_2_/FiO_2_ ratio in patients with higher PaO_2_. However, statistical significance was not achieved at any time point. In a similar study comparing conservative (PaO_2_ of 70-100 mm Hg and oxygen saturation {SpO_2_} of 94%-98%) to conventional (PaO_2_ up to 150 mm Hg and SpO_2_ of 97%-100%) oxygen therapy in ICU patients, the daily average FiO_2_ requirements were found to be higher in the conventional group as compared to the conservative group [5]. In our study, there was no statistically significant difference in terms of FiO_2_ and PaO_2_/FiO_2_ between the two groups due to a small PaO_2_ difference among the two groups and because of a smaller sample size of the records analyzed.

We have used the SOFA score to assess the severity of organ dysfunction in COVID-19 patients. SOFA scoring system was initially designed for critically ill patients with sepsis [6,7]. It is a scoring tool to evaluate organ dysfunction using six reproducible organ system variables: circulation, respiration, liver, renal, central nervous system, and coagulation function [6,7]. Although there were concerns regarding COVID-19 pneumonia mainly affecting the respiratory system and questions were being raised regarding the validity of SOFA score as a prognostic indicator in these patients, multiple studies have shown that the disease more than just affects the respiratory system and SOFA scoring can be used as an important prognostic tool in these groups of patients [8-10]. Recent studies have concluded that SARS-CoV-2 infection can cause significant lung injury, as well as damage to the skin, blood system, endocrine system, neurological system, kidney, liver, and heart, which could all lead to skin lesions, hyperglycemic state, gastrointestinal manifestations, thrombotic state, acute coronary syndromes, and arrhythmia [11,12].

In our study, the baseline SOFA score and the SOFA score at 48 hours were found to be significantly higher in the low PaO_2_ group compared to the high PaO_2_ group. This could be explained by the fact that the patients in the low PaO_2_ group might have already been affected more severely by the disease, resulting in a higher SOFA score. However, the change in SOFA score over 48 hours between the two groups was not statistically significant (p = 0.257). Similar findings were noted in a study by Gelissen et al., where they found that the SOFA_RANK_ score (SOFA score excluding the respiratory component) in patients with non-respiratory organ failure was not significantly different in the low and high PaO_2_ groups [13]. However, a study by Yang et al. concluded that flow oxygen therapy is an independent protective factor in COVID-19 patients [14].

In a systemic review and meta-analysis conducted by Chu et al., it was concluded that liberal oxygen therapy leads to significantly poorer outcomes and higher mortality in patients who had acute severe illnesses while not showing any improvement in other parameters [15]. They demonstrated an unfavorable outcome if patients were given supplemental oxygen at an SpO_2_ of 94%-96%. In another meta-analysis conducted by Barbateskovic et al., it was concluded that there was very low certainty evidence of harm that patients undergoing oxygen therapy with high FiO_2_ faced at a three-month endpoint compared to patients undergoing low FiO_2_ [16]. There was no statistically significant difference in the incidence of lung injuries between patients undergoing oxygen therapy with high FiO_2_ at a three-month endpoint compared to patients undergoing low FiO_2_.

A previous study done by Iepsen et al. suggested that patients with severe COVID-19 exhibit near-normal blood lactate, indicating preserved mitochondrial function, despite a systemic inflammatory state similar to sepsis [17]. Fridman et al. found that the serum lactate practically remains near normal throughout the disease in COVID-19 pneumonia [18]. Our study showed similar results where the serum lactate level was variable but not progressive through the course of the disease and did not differ significantly between the two groups at various time points.

The mortality rate was higher in the high PaO_2_ group than in the low PaO_2_ group. However, the two groups had no statistically significant difference in mortality (p = 0.485). That was against the study conducted by Girardis et al., where they stated that oxygen supplemented to a more conservative oxygen saturation target (94%-98%) was associated with improved outcomes in terms of mortality compared with conventional oxygen administration in which PaO_2_ was significantly higher [5]. They found an absolute mortality reduction of 8.6% in the conservative oxygen group. In a study conducted by Barrot et al. for liberal versus conservative oxygen therapy in patients with ARDS, they found a clinically relevant excess of mortality in the conservative oxygen group, with mortality being 14% higher than that in the liberal oxygen group at 90 days. They suggested that although decreasing oxygen exposure could decrease lung damage in the early phase of the disease, patients were predisposed to hypoxia. Moreover, they had to terminate their trial prematurely due to increased mortality at 90 days and mesenteric ischemia in the conservative group [19]. Two other recent randomized controlled trials (RCTs) failed to show any benefit of a conservative oxygenation strategy in improving mortality [20,21]. Our study also found a statistically non-significant difference between the two groups in terms of mortality.

Seven out of 30 patients were extubated in the high PaO_2_ group, while two out of 23 patients were extubated in the low PaO_2_ group. There was no statistically significant difference in the rate of extubation within 48 hours between the two groups (p = 0.485). A meta-analysis by Lee et al. showed no statistical difference in mortality between early and late intubation [22].

The incidence of AKI in our study was found to be comparable between the two groups (p = 0.8), suggesting that either the conservative or liberal use of oxygen therapy did not help in decreasing the incidence of AKI in COVID-19-infected patients. Acute kidney injury is a frequent complication seen in patients suffering from COVID-19 ARDS, especially those requiring ICU admission. Some commonly cited hypotheses for this include acute tubular necrosis caused by tubular epithelial and podocyte damage by the virus, the direct infection of glomerular endothelia, COVID-19-related hypovolemia leading to prerenal AKI, complement activation, cytokine storm, hypercoagulability, and nephrotoxic drugs [23]. AKI is considered an independent risk factor for increased mortality in critically ill patients of any disease, including COVID-19. Kidney involvement has also been reported as an indicator of poor prognosis regardless of initial COVID-19 severity [24,25]. Charytan et al., in their study, found that patients with AKI were more likely to be admitted to the ICU, undergo mechanical ventilation, or undergo extracorporeal membrane oxygenation during admission than those without AKI. Among individuals with an outcome (death or discharge), mortality was higher than without AKI [26].

The change in the Glasgow Coma Scale over 48 hours in our study was comparable between the two groups. Fällmar et al., in their study, showed that the neuroradiological findings in COVID-19 are correlated to blood biomarkers, the Glasgow Coma Scale, and days in the intensive care unit [27].

Our study had certain limitations. Firstly, it was a retrospective study. So, we could not precisely target the PaO_2_ values in either of the groups. However, we tried to include only those records where ABG values were available and had stayed in the desired range for a period of 48 hours. Secondly, the sample size was very low. That may be the reason for some of the non-significant results in our study. So, more detailed studies considering these parameters and with larger sample sizes are required to decide regarding the optimal target of partial pressure of oxygen while considering oxygen therapy for COVID-19-infected patients.

## Conclusions

Our study highlights that targeting either low or high arterial oxygen content while considering oxygen therapy for COVID-19 patients did not significantly alter the outcomes. However, prospective studies with higher sample sizes are required to decide the optimal PaO_2_ in COVID-19 patients.
